# Supplementary material for: Natural Killer cells demonstrate distinct eQTL and transcriptome-wide disease associations, highlighting their role in autoimmunity
Source: Nat Commun. 2022 Jul 14;13:4073. doi: 10.1038/s41467-022-31626-4 (PMC9283523; doi:10.1038/s41467-022-31626-4)

Natural Killer cells demonstrate distinct eQTL and transcriptome-wide disease associations, highlighting their role in autoimmunity

James J Gilchrist[1,2,3†*], Seiko Makino[3*], Vivek Naranbhai[3*], Piyush K Sharma[2,4]
Surya Koturan[2,4], Orion Tong[2,4], Chelsea A Taylor[2,4] Robert A Watson[2,4]
Alba Verge de los Aires[2,4], Rosalin Cooper[2,4], Evelyn Lau[3], Sara Danielli[3] Dan Hameiri-Bowen[3]
Wanseon Lee[3], Esther Ng[3], Justin Whalley[3], Julian C Knight[3,5†*], Benjamin P Fairfax[2,4,6†*]

[1]Department of Paediatrics, University of Oxford, Oxford, UK
[2]MRC–Weatherall Institute of Molecular Medicine, University of Oxford, Oxford, UK
[3]Wellcome Centre for Human Genetics, University of Oxford, Oxford, UK
[4]Department of Oncology, University of Oxford, Oxford, UK
[5]Chinese Academy of Medical Science Oxford Institute, University of Oxford, Oxford, UK [6]NIHR Oxford Biomedical Research Centre, Oxford University Hospitals NHS Foundation Trust, Oxford, UK
*Equal contribution
† corresponding

Supplementary Information

Supplementary Figure 1: The effects of principal component incorporation for *cis* and *trans* eQTL mapping.

Supplementary Figure 2: Gene expression in NK cells and sharing of eQTL between NK cells and other primary immune cells.

Supplementary Figure 3: Functional annotation of NK cell eQTL.

Supplementary Figure 4: GOBP enrichment of NK cell-specific eQTL.

Supplementary Figure 5: Effect of background GWAS trait on NK cell eQTL GWAS enrichment.

Supplementary Figure 6: NK cell eQTL at *ERAP2*.

Supplementary Figure 7: NK cell specific Allele specific expression at *MC1R*.

Supplementary Figure 8: Study power.

Supplementary Figure 9: Study sample population structure.

Supplementary Figure 10: NK cell immunophenotyping.

Supplementary Figure 1: The effects of principal component incorporation for *cis* and *trans* eQTL mapping.
Graphs demonstrate the effect of incorporating between 0 and 50 principal components as covariates on eQTL discovery in *cis* (top) and *trans* (bottom) mapping. The number of eQTLs discovered represent approximate passes, and actual numbers differ in the final analysis.

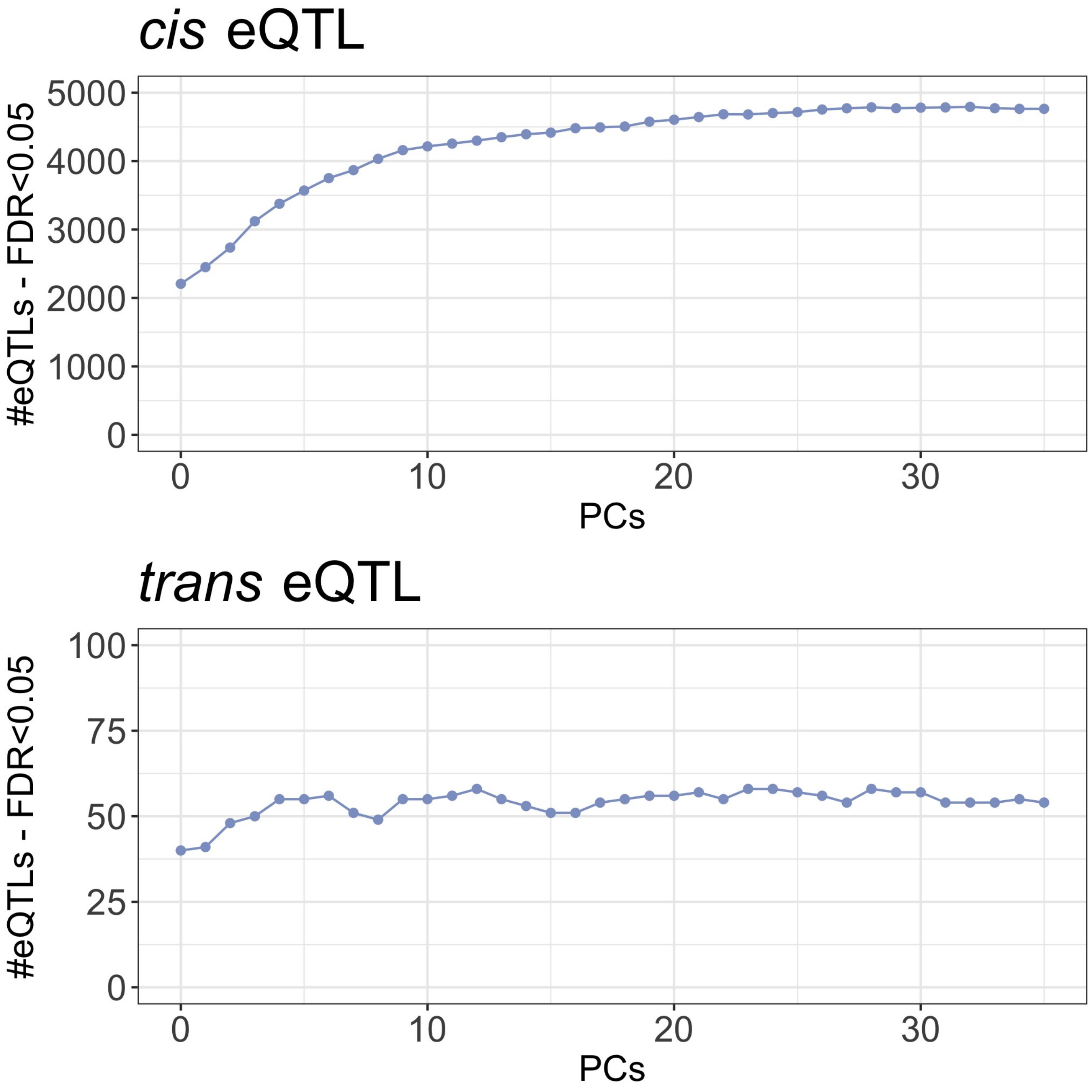

Supplementary Figure 2: Gene expression in NK cells and sharing of eQTL between NK cells and other primary immune cells.
Box plot depicting median expression in NK cells for genes (n=2,161) with eQTL in NK cells that are shared with at least one other primary immune cell subsets (neutrophils, monocytes, $CD4^+$ T cells, $CD8^+$ T cells), and genes with eQTL that are unique to NK cells (n=588). Box and whisker plot; boxes depict the upper and lower quartiles of the data, and whiskers depict the range of the data excluding outliers (outliers are defined as data-points > 1.5× the inter-quartile range from the upper or lower quartiles). Expression between groups were compared with Mann-Whitney U-tests. P-values are two-sided.

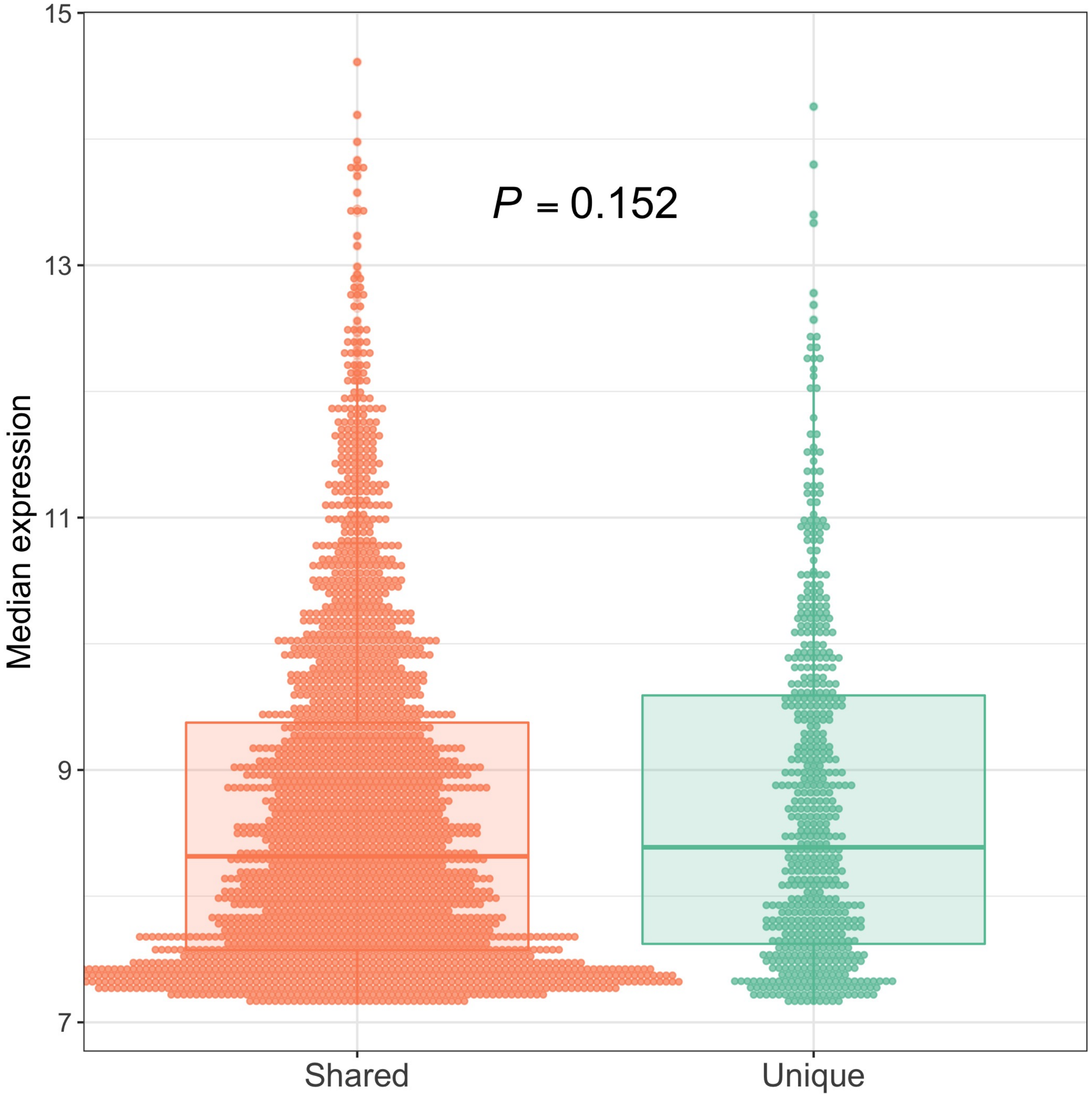

Supplementary Figure 3: Functional annotation of NK cell eQTL.
Frequency plots representing the density of eQTL loci surrounding functional genomic tracks. P-values (permutation) represent enrichment; significantly overrepresented features are coloured pink, underrepresented features blue. Densities are plotted for primary eQTL (A), secondary eQTL (B) and NK cell-specific eQTL (C). Enrichment of eQTL loci at transcription factor binding sites for primary eQTL (D), secondary eQTL (E) and NK cell-specific eQTL (F). Significantly enriched transcription factor binding sites are highlighted (pink). (G) Comparison of functional track and transcription factor binding sites overlap observed in primary and secondary eQTL. P-values calculated with Fisher Exact tests. (H) Comparison of functional track and transcription factor binding sites overlap observed in eQTL specific to NK cells eQTL shared with other cell types. Significantly overrepresented features are coloured pink, underrepresented features blue.

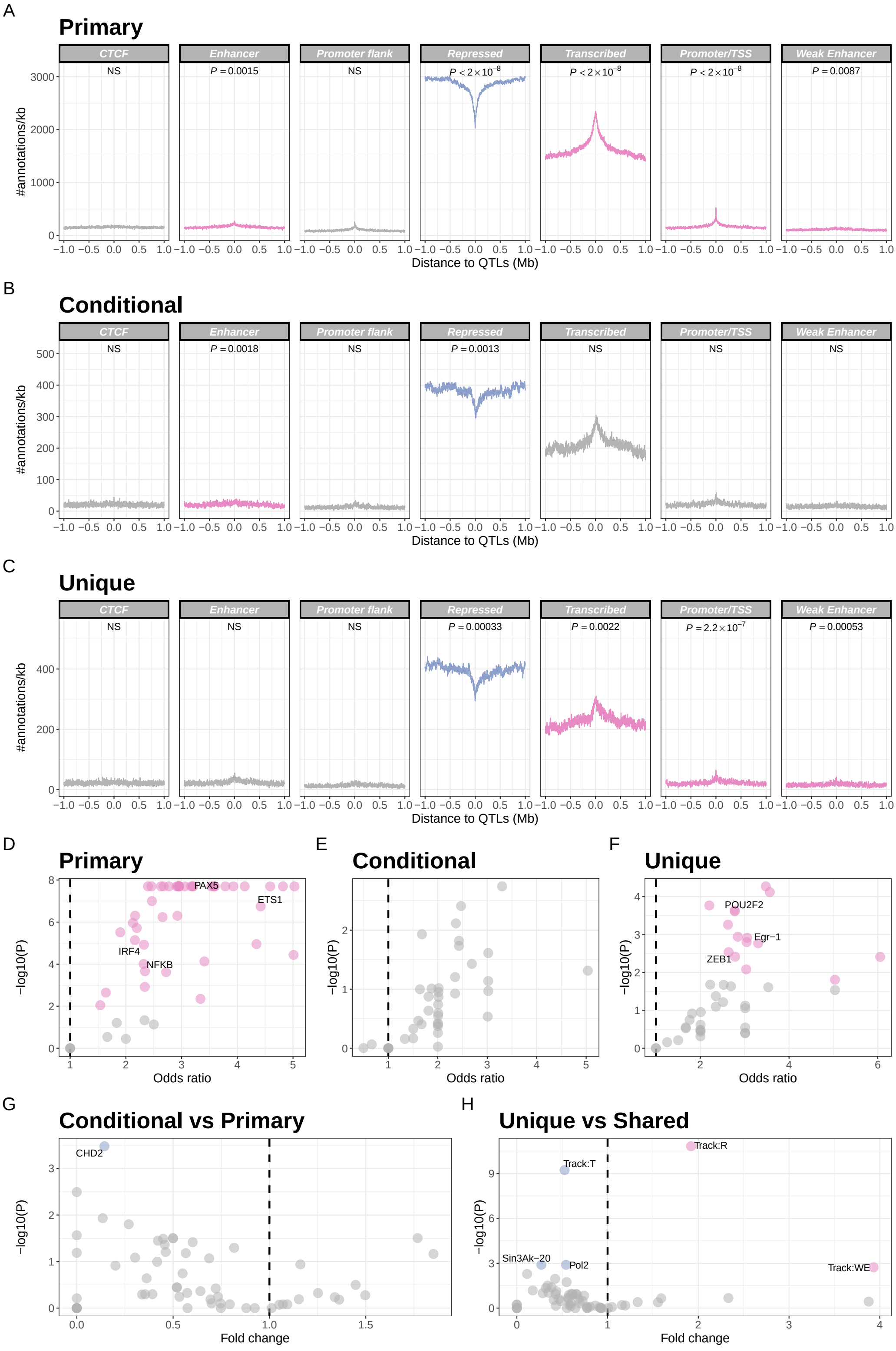

Supplementary Figure 4: GOBP enrichment of NK cell-specific eQTL.
GOBP pathway analysis of genes with eQTL specific to NK cells (as compared with monocytes, neutrophils, $CD4^+$ and $CD8^+$ T cells. All depicted pathways are significantly enriched ($FDR < 0.05$). nOverlap, number of overlapping genes from a pathway. Enrichment is calculated using a hypergeometric test.

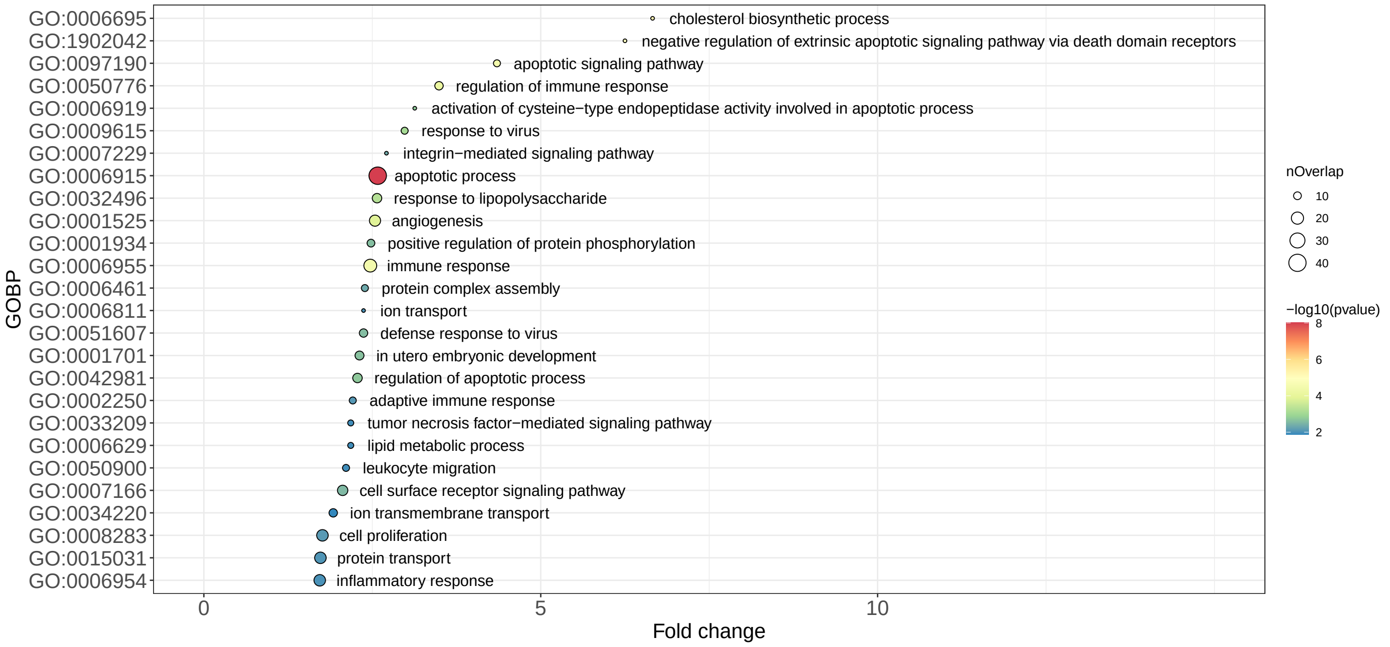

Supplementary Figure 5: Effect of background GWAS trait on NK cell eQTL GWAS enrichment. Comparison of observed enrichment of shared causal loci between NK cell eQTL and GWAS traits (n=100) according to background trait. Traits are compared with the enrichment observed for height (x-axis) and whole body impedence (y-axis) as background. Significant traits ($FDR < 0.05$ using height as background) are highlighted (pink). Point size is proportional to a trait's number of GWAS-significant loci.

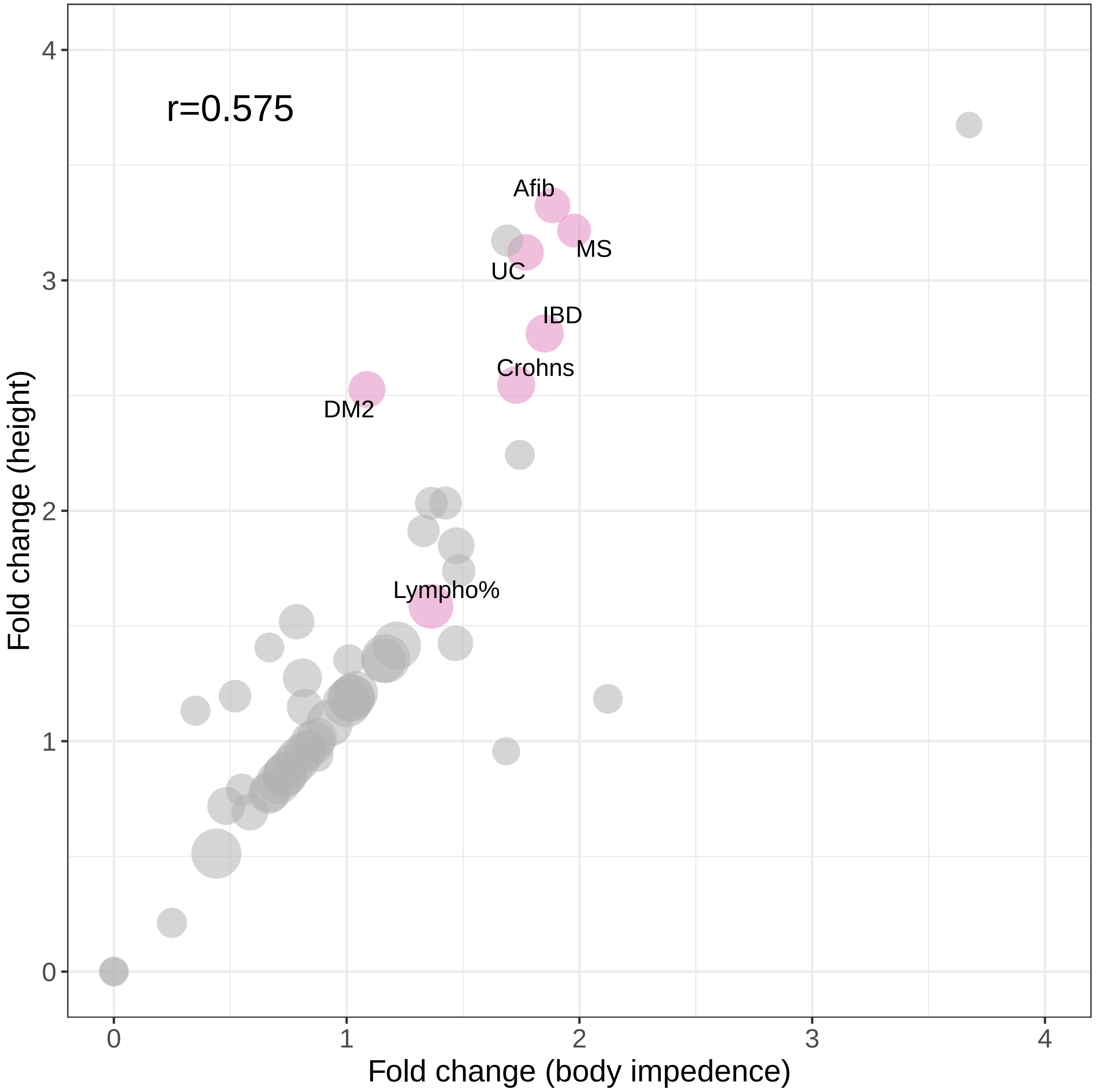

Supplementary Figure 6: NK cell eQTL at *ERAP2*.
(A) Effect of rs1363974 genotype on *ERAP2* expression in NK cells (n=245 individuals). Box and whisker plot; boxes depict the upper and lower quartiles of the data, and whiskers depict the range of the data excluding outliers (outliers are defined as data-points > 1.5× the inter-quartile range from the upper or lower quartiles) Genotype to phenotype correlations were calculated with linear regression. P-values are two-sided. (B) The effect of rs1363974 genotype on *ERAP2* expression is common to NK cells, monocytes, neutrophils, $CD4^+$ and $CD8^+$ T cells. The *ERAP2* eQTL in NK cells colocalises with a genetic locus which determines neutrophil percentage (C) and lymphocyte percentage (D). SNPs are coloured according to strength of LD (CEU population) to the peak eSNP (rs1363974); brown $r^2 > 0.8$, orange $0.5 < r^2 \leq 0.8$, yellow $0.2 < r^2 \leq 0.5$, grey $r^2 leq 0.2$.

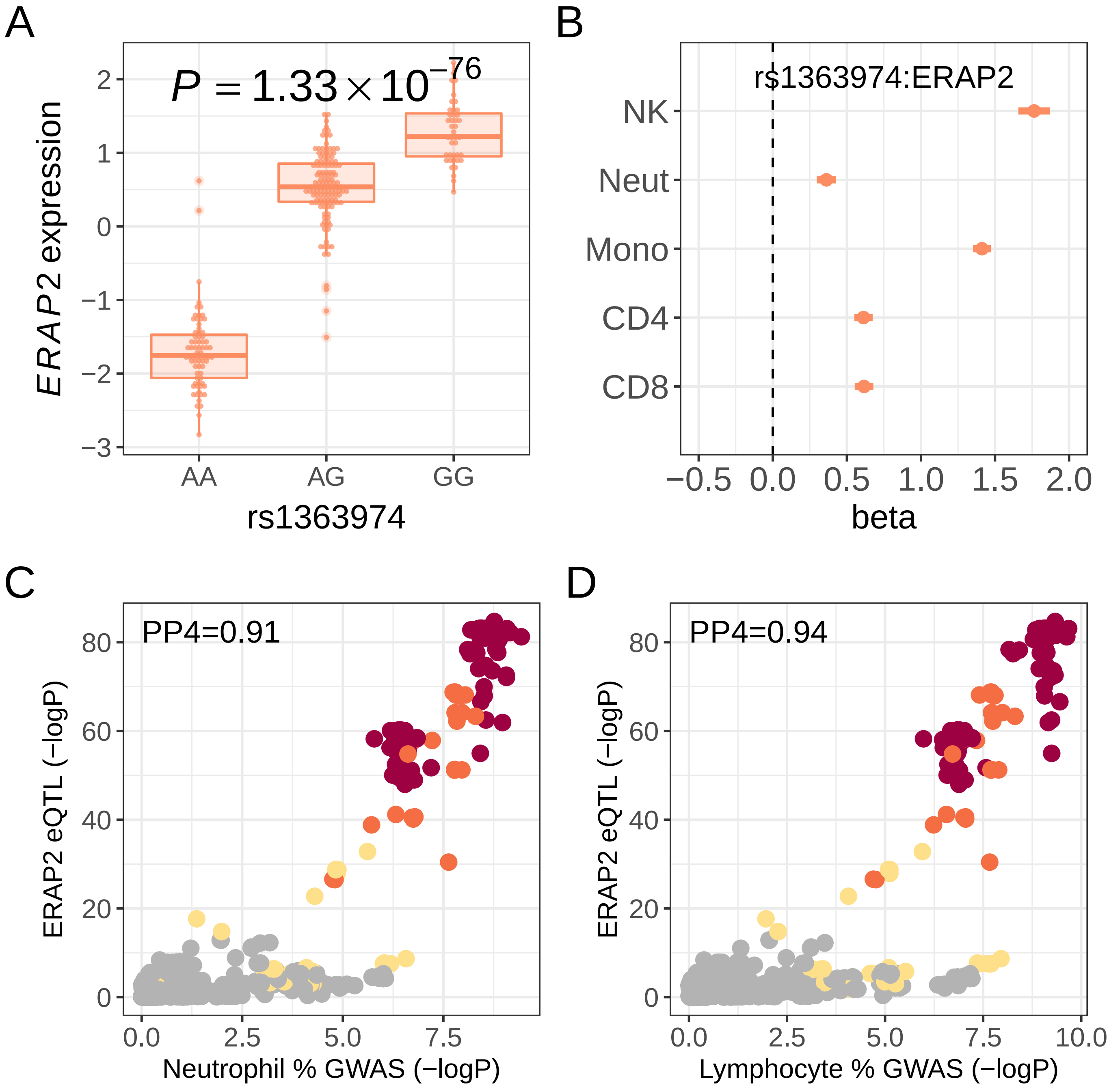

Supplementary Figure 7: NK cell specific Allele specific expression at *MC1R*.
Allele specific expression was measured at the SNP rs2228479, in R^2 of 1 with the peak *cis* eQTL at this gene, rs117406136 across cDNA from individuals heterozygote at this allele using the C-BASE assay in monocytes from 5 individuals, where no difference was seen, and in NK cells. Significance of effect for NK cells was performed using a χ^2 (1 degree of freedom), comparing ratios of genomic to cDNA. A paired t-test was performed for the monocyte samples. Exact p-value for comparison of NK cell cDNA:gDNA proportions: $p = 2.37 \times 10^{-37}$.

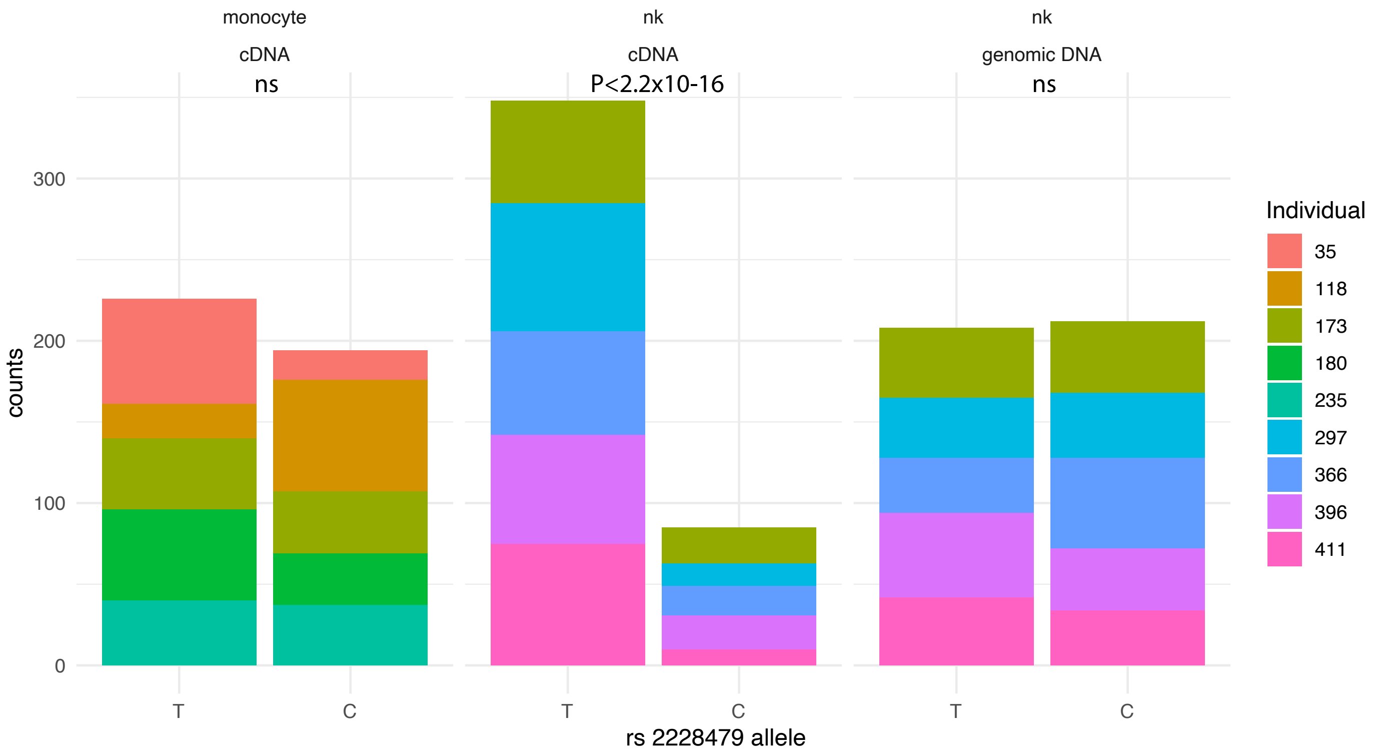

Supplementary Figure 8: Study power.
Study power for eQTL discovery in *cis* and *trans*. For *cis* power calculations we assume a variable number of independent tests, defined as the number of independent SNPs in a cis testing window (1-5,000) multiplied by the number of genes test (18,000). For *trans* power calculations we assume 18e9 independent tests, assuming 18,000 genes and 1e6 independent SNPs genome-wide. In each case we calculate power for $alpha = 0.05$/number of tests and an effect size and standard deviation of 0.13.

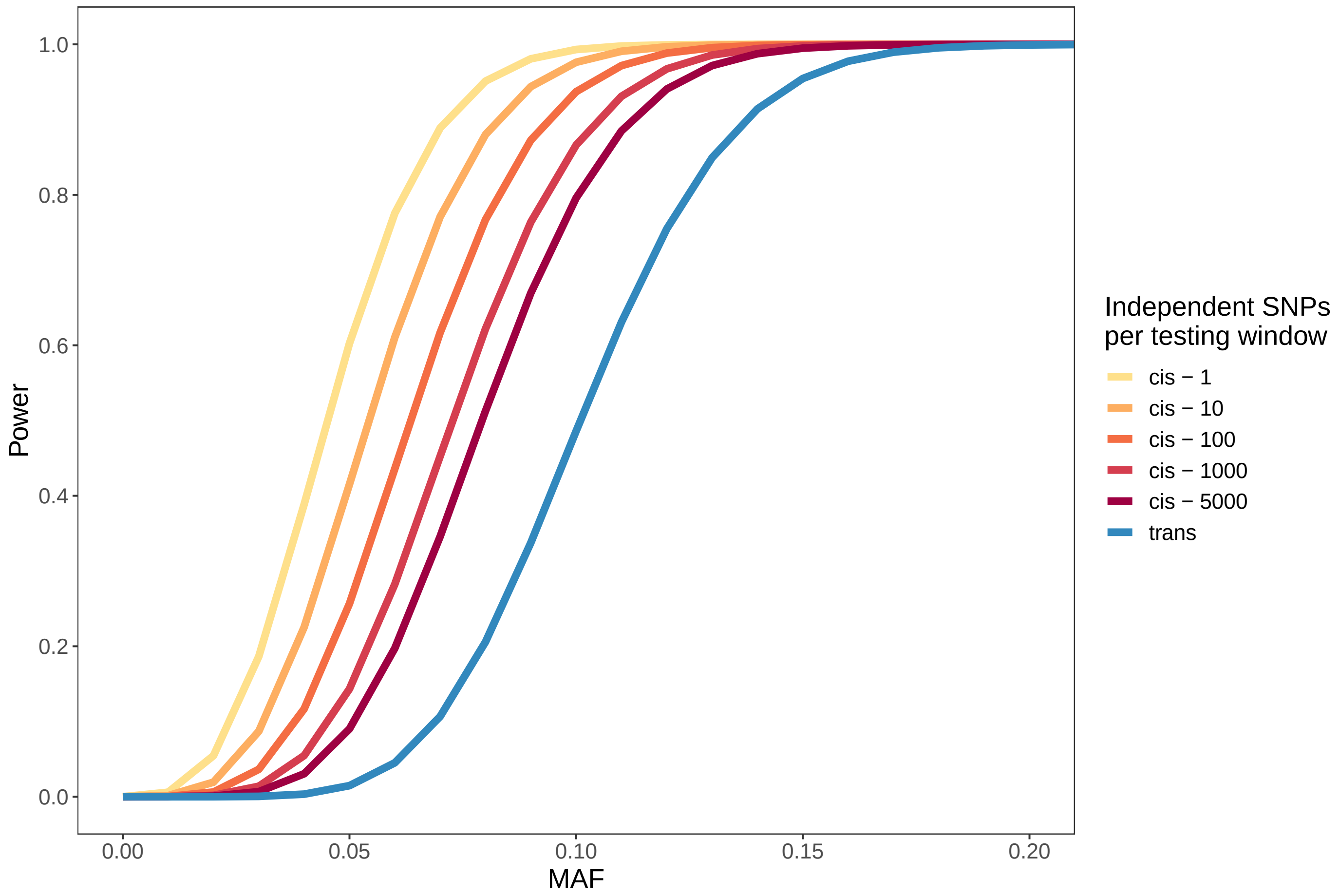

Supplementary Figure 9: Study sample population structure.
Plot of the major two principal components of genome wide genotyping data. Study samples are plotted in blue against a background of 1000G project samples (coloured according to super-population membership: AFR, African, AMR, American; EAS, East Asian; EUR, European; SAS, South Asian).

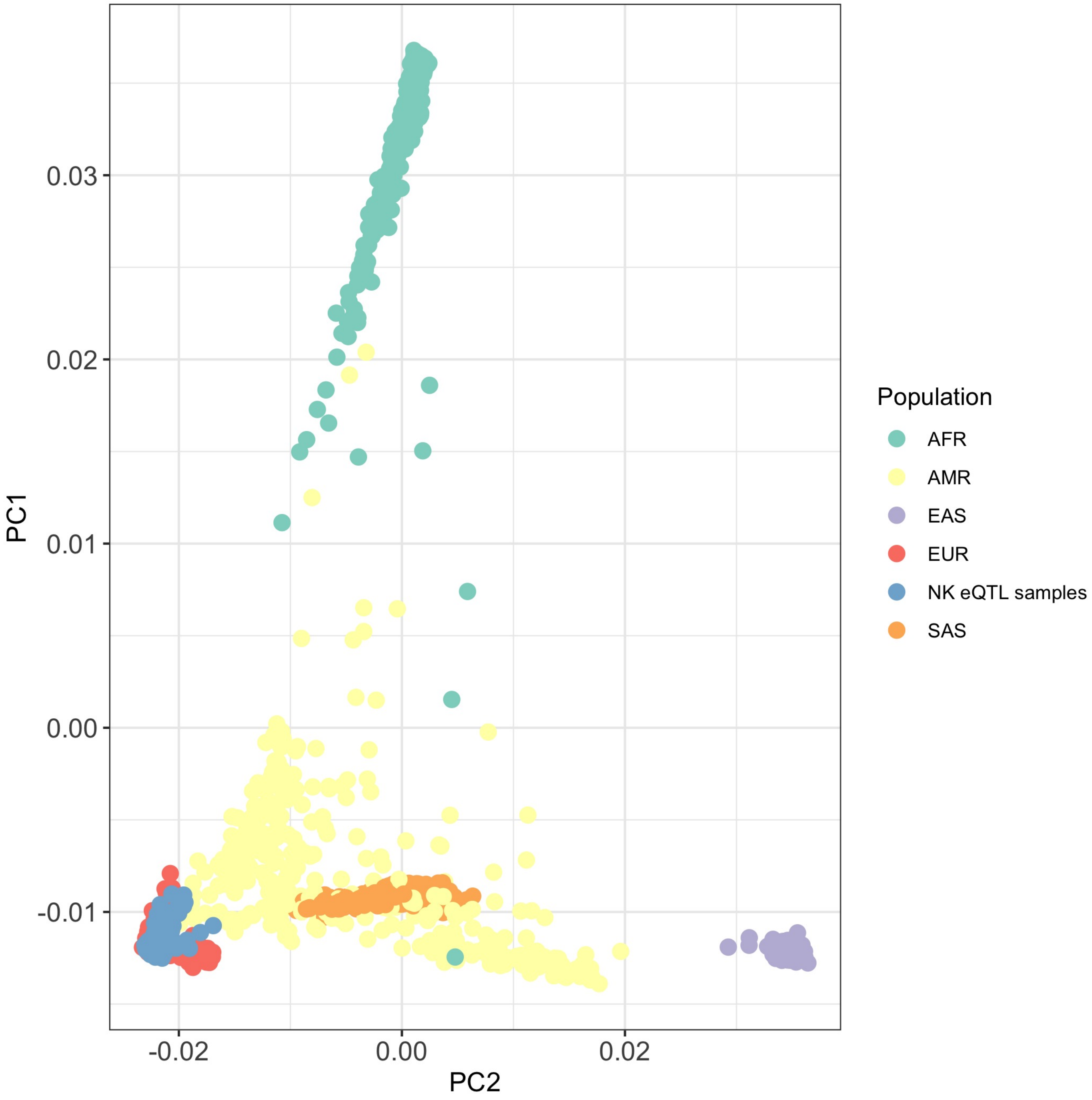

Supplementary Figure 10: NK cell immunophenotyping.
Gating strategy to define CD226$^+$, CD57$^+$ and KIR2D$^+$ NK cell populations in metastatic melanoma patients (see Figure 4). Flow cytometry was performed using a BD LSR Fortessa X20 flow cytometer, and the data analysed using FlowJo v10.8 Software (BD Life Sciences).

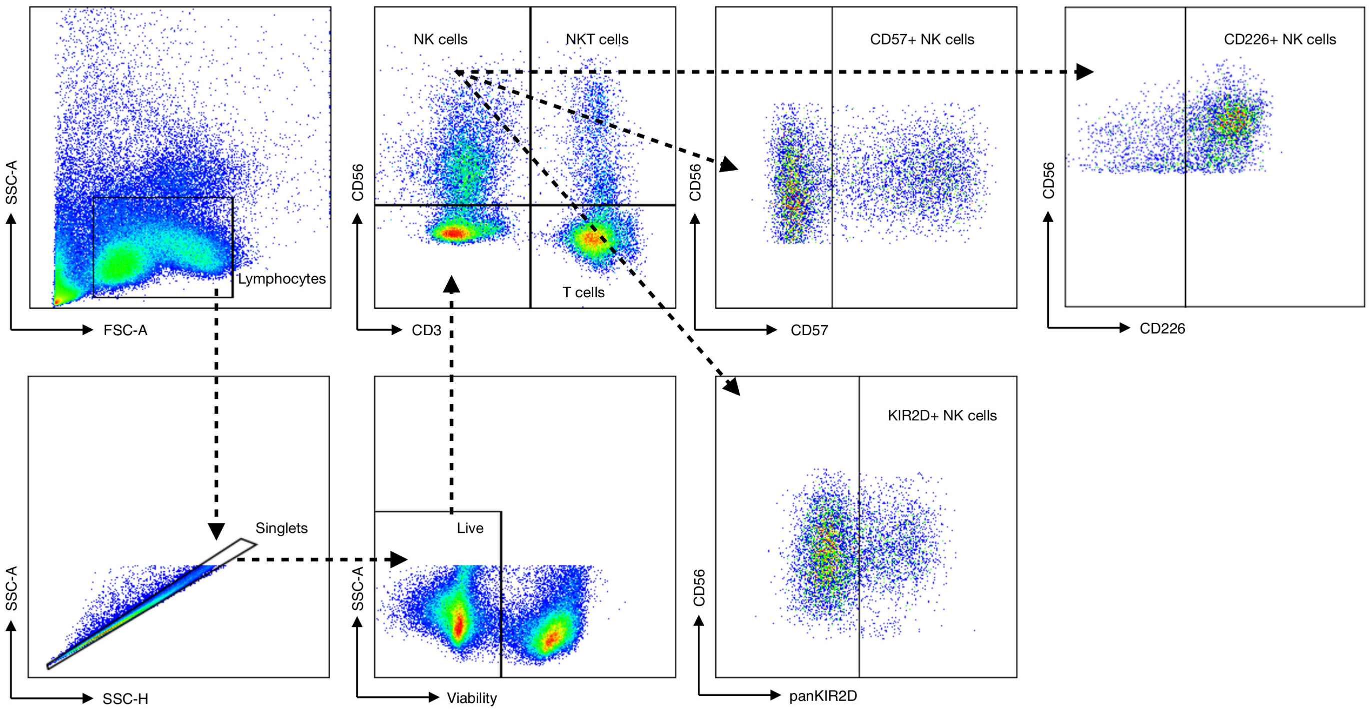

Supplement: Supplementary file 1 — Supplementary Information [file 41467_2022_31626_MOESM1_ESM.pdf]
