# Supplementary material for: Natural Killer cells demonstrate distinct eQTL and transcriptome-wide disease associations, highlighting their role in autoimmunity
Source: Nat Commun. 2022 Jul 14;13:4073. doi: 10.1038/s41467-022-31626-4 (PMC9283523; doi:10.1038/s41467-022-31626-4)
Supplement: Supplementary file 3 — Description of Additional Supplementary Files [file 41467_2022_31626_MOESM3_ESM.pdf]

Description of Additional Supplementary Files

File Name: Supplementary Data 1
Description: Peak *cis* eSNP association statistics at 3,951 *cis* eQTLs in primary NK cells.

File Name: Supplementary Data 2
Description: Peak *cis* eSNP association statistics at 4,545 significant primary and conditional *cis* eQTLs in primary NK cells, with RTC GWAS trait overlap at each *cis* eQTL.

File Name: Supplementary Data 3
Description: Sharing of *cis* eQTL in NK cells with primary immune cells (monocyte, B cells, $CD4^+$ and $CD8^+$ T cells), overlap with ENCODE features and GWAS traits (Regulatory Trait Concordance and coloc).

File Name: Supplementary Data 4
Description: Evidence for enrichment of ENCODE features among *cis* eQTL in NK cells.

File Name: Supplementary Data 5
Description: Evidence for enrichment of GOBP terms among *cis* eQTL in NK cells.

File Name: Supplementary Data 6
Description: Regulatory Trait Concordance results for *cis* eQTL in NK cells.

File Name: Supplementary Data 7
Description: Evidence for enrichment of GWAS signal colocalization for GWAS Catalog (n=60) and UK Biobank (n=40) traits with NK cell *cis* eQTLs.

File Name: Supplementary Data 8
Description: Peak *trans* eSNP association statistics at 84 *trans* eQTLs in primary NK cells.

File Name: Supplementary Data 9
Description: KIR*IMP imputation accuracy.

File Name: Supplementary Data 10
Description: Significant KIR type *trans* eQTL associations in NK cells.

File Name: Supplementary Data 11
Description: Transcriptome-wide association study statistics of NK cell gene expression in ulcerative colitis.

File Name: Supplementary Data 12
Description: Transcriptome-wide association study statistics of NK cell gene expression in rheumatoid arthritis.

File Name: Supplementary Data 13
Description: Transcriptome-wide association study statistics of NK cell gene expression in systemic lupus erythematosus.

File Name: Supplementary Data 14
Description: Transcriptome-wide association study statistics of NK cell gene expression in primary biliary cirrhosis.

File Name: Supplementary Data 15
Description: Transcriptome-wide association study statistics of NK cell gene expression in Crohn’s disease.

File Name: Supplementary Data 16
Description: GWAS studies/traits from the NHGRI-EBI GWAS Catalog used for Regulatory Trait Concordance analysis.
