# Supplementary material for: Natural Killer cells demonstrate distinct eQTL and transcriptome-wide disease associations, highlighting their role in autoimmunity
Source: Nat Commun. 2022 Jul 14;13:4073. doi: 10.1038/s41467-022-31626-4 (PMC9283523; doi:10.1038/s41467-022-31626-4)
Supplement: Supplementary file 5 — Reporting Summary [file 41467_2022_31626_MOESM5_ESM.pdf]

nature portfolio

Corresponding author(s): James Gilchrist, Julian Knight, Benjamin Fairfax

Last updated by author(s): 13th May 2022

Reporting Summary

Nature Portfolio wishes to improve the reproducibility of the work that we publish. This form provides structure for consistency and transparency in reporting. For further information on Nature Portfolio policies, see our Editorial Policies and the Editorial Policy Checklist.

Statistics

For all statistical analyses, confirm that the following items are present in the figure legend, table legend, main text, or Methods section.

n/a	Confirmed	
☐	☒	The exact sample size (*n*) for each experimental group/condition, given as a discrete number and unit of measurement
☐	☒	A statement on whether measurements were taken from distinct samples or whether the same sample was measured repeatedly
☐	☒	The statistical test(s) used AND whether they are one- or two-sided<br>*Only common tests should be described solely by name; describe more complex techniques in the Methods section.*
☐	☒	A description of all covariates tested
☐	☒	A description of any assumptions or corrections, such as tests of normality and adjustment for multiple comparisons
☐	☒	A full description of the statistical parameters including central tendency (e.g. means) or other basic estimates (e.g. regression coefficient) AND variation (e.g. standard deviation) or associated estimates of uncertainty (e.g. confidence intervals)
☐	☒	For null hypothesis testing, the test statistic (e.g. *F*, *t*, *r*) with confidence intervals, effect sizes, degrees of freedom and *P* value noted<br>*Give P values as exact values whenever suitable.*
☒	☐	For Bayesian analysis, information on the choice of priors and Markov chain Monte Carlo settings
☒	☐	For hierarchical and complex designs, identification of the appropriate level for tests and full reporting of outcomes
☐	☒	Estimates of effect sizes (e.g. Cohen's *d*, Pearson's *r*), indicating how they were calculated

Our web collection on statistics for biologists contains articles on many of the points above.

Software and code

Policy information about availability of computer code

Data collection	Flow cytometry data was collected using FlowJo version 10.
Data analysis	Scripts and source data used to produce figures, alongside scripts used in data analysis are available at: https://github.com/jjgilchrist/NK_eQTL.<br><br>QTL mapping was performed with QTLtools (v1.3.1). Genotypes were phased and imputed using SHAPEIT (v2.17) and IMPUTE2 (v2.3.2). KIR copy number imputation was performed with KIR*IMP v1.2.0.<br><br>R version 3.6.3, R packages; lumi (v2.48.0), sva (v3.44.0), coloc v5.1.0, XGR (v1.1.8), ggplot2 (v3.0.0).

For manuscripts utilizing custom algorithms or software that are central to the research but not yet described in published literature, software must be made available to editors and reviewers. We strongly encourage code deposition in a community repository (e.g. GitHub). See the Nature Portfolio guidelines for submitting code & software for further information.

Data

Policy information about availability of data

All manuscripts must include a data availability statement. This statement should provide the following information, where applicable:

- Accession codes, unique identifiers, or web links for publicly available datasets
- A description of any restrictions on data availability
- For clinical datasets or third party data, please ensure that the statement adheres to our policy

Sample genotypes are available at the European Genome-Phenome Archive (EGA) with accession ID EGAS00000000109: https://ega-archive.org/studies/EGAS00000000109. Raw gene expression data and probe QC filters have been deposited at: https://doi.org/10.5281/zenodo.6352656. Summary statistics are available via R Shiny, interactive, browser-based applications for cis mapping data (https://jjgilchrist.shinyapps.io/nk_cis_eqtl/) and for trans mapping data (https://jjgilchrist.shinyapps.io/nk_trans_eqtl/). We have also contributed raw genotype and phenotype data to the eQTL Catalogue project (https://www.ebi.ac.uk/eqtl/).

The study makes use of the following publicly-available data; Gene Ontology consortium (http://www.geneontology.org), ENCODE Project (https://www.encodeproject.org), Open Targets Genetics (https://genetics.opentargets.org). UK Biobank summary statistics (http://www.nealelab.is/uk-biobank/), , eQTLGEN Consortium (https://www.eqtlgen.org).

Field-specific reporting

Please select the one below that is the best fit for your research. If you are not sure, read the appropriate sections before making your selection.

☒ Life sciences ☐ Behavioural & social sciences ☐ Ecological, evolutionary & environmental sciences

For a reference copy of the document with all sections, see nature.com/documents/nr-reporting-summary-flat.pdf

Life sciences study design

All studies must disclose on these points even when the disclosure is negative.

Sample size	245 donors were included in the study. We estimate that our study has 80% power to detect a cis eQTL with a causal eSNP with minor allele frequency >0.08, assuming 100 independent tests in the cis testing window. In trans, our study has 80% power to detect a trans eQTL with a causal eSNP with minor allele frequency >0.12.
Data exclusions	No samples were excluded from the analysis.
Replication	We assessed evidence for replication of our trans eQTL using eQTLGEN consortium summary statistics. Replication was also attempted for three genes displaying eQTL at the protein level using flow cytometry in a separately recruited cohort of patients with metastatic melanoma.
Randomization	The study seeks to correlate genotype with RNA expression in immune cells, and so randomization is not appropriate.
Blinding	All sample analysis was performed blinded to genotype.

Reporting for specific materials, systems and methods

We require information from authors about some types of materials, experimental systems and methods used in many studies. Here, indicate whether each material, system or method listed is relevant to your study. If you are not sure if a list item applies to your research, read the appropriate section before selecting a response.

Materials & experimental systems

n/a	Involved in the study	
☐	☒	Antibodies
☒	☐	Eukaryotic cell lines
☒	☐	Palaeontology and archaeology
☒	☐	Animals and other organisms
☐	☒	Human research participants
☒	☐	Clinical data
☒	☐	Dual use research of concern

Methods

n/a	Involved in the study	
☒	☐	ChIP-seq
☐	☒	Flow cytometry
☒	☐	MRI-based neuroimaging

Antibodies

Antibodies used	After staining with viability dye (LIVE/DEAD FixableNear-IR Dead Cell Stain cat# L10119, Thermo-Fisher Scientific) at a concentration of 1:1000 in PBS, cells were surface immunostained with BUV496-conjugated anti-CD3 (1:150 dilution, clone UCHT1, cat# 612940, BD Biosciences), BUV395-conjugated anti-CD56 (1:50 dilution, clone NCAM16.2, cat# 563555, BD Biosciences), VioGreen-conjugated

	PanKIR2D (1:50 dilution, cat# 130-128-216, Miltenyi Biotec), BV785-conjugated anti-CD57 (1:50 dilution, clone QA17A04, cat# 393329, Biolegend) and PE-conjugated anti-CD226 (1:100 dilution, clone 11A8, cat# 338305, Biolegend).
Validation	As per manufacturers: https://www.bdbiosciences.com/, https://www.miltenyibiotec.com/, https://www.biolegend.com/. All antibodies validated by manufacturer for flow cytometry of human cells.

Human research participants

Policy information about studies involving human research participants

Population characteristics	Healthy individuals of European ancestry were included in the study. Participants had a median age of 28 years (range 18-66) and 117 of 245 were male.
Recruitment	Healthy donors were recruited following written informed consent through the Oxford Biobank (www.oxfordbiobank.org.uk) . Metastatic melanoma patients following written informed consent through the Oxford Radcliffe Biobank during routine clinical care for melanoma.
Ethics oversight	Oxfordshire Research Ethics Committee COREC reference 06/Q1605/55 (healthy donors). Oxford Centre for Histopathology Research ethical approval nos. 16/A019 and 18/A064 (melanoma patients).

Note that full information on the approval of the study protocol must also be provided in the manuscript.

Flow Cytometry

Plots

Confirm that:

☒ The axis labels state the marker and fluorochrome used (e.g. CD4-FITC).

☒ The axis scales are clearly visible. Include numbers along axes only for bottom left plot of group (a 'group' is an analysis of identical markers).

☒ All plots are contour plots with outliers or pseudocolor plots.

☒ A numerical value for number of cells or percentage (with statistics) is provided.

Methodology

Sample preparation	PBMCs were prepared, frozen and stored in liquid Nitrogen in 90%FBS/10% DMSO for later use in flow. Samples were thawed and immunostained with antibodies as detailed above. Cells were stained in HBSS containing 5% fetal calf serum on ice and in the dark for 30 minutes, then fixed in 2% paraformaldehyde. All samples included fixable amine reactive viability dye.
Instrument	LSR II
Software	FlowJo version 10
Cell population abundance	NA
Gating strategy	Supplementary Figure 10.

☒ Tick this box to confirm that a figure exemplifying the gating strategy is provided in the Supplementary Information.
